# 3D Motion Estimation of Left Ventricular Dynamics Using MRI and Track-to-Track Fusion

**DOI:** 10.1109/JTEHM.2020.2989390

**Published:** 2020-04-24

**Authors:** Kumaradevan Punithakumar, Ismail Ben Ayed, Abraam S. Soliman, Aashish Goela, Ali Islam, Shuo Li, Michelle Noga

**Affiliations:** 1 Department of Radiology and Diagnostic ImagingUniversity of Alberta 3158 EdmontonABT6G 2R3Canada; 2 Servier Virtual Cardiac CentreMazankowski Alberta Heart Institute 103114 EdmontonABT6G 2B7Canada; 3 Department of Computing ScienceUniversity of Alberta 3158 EdmontonABT6G 2R3Canada; 4 École de Technologie Supérieure (ÉTS)MontrealQCH3C 1K3Canada; 5 MR R&DPhilips Healthcare5684 PCBestThe Netherlands; 6 Department of Medical ImagingWestern UniversityLondonONN6A 3K7Canada; 7 St. Joseph’s Health Care LondonLondonONN6A 4V2Canada

**Keywords:** Multiview fusion, cardiac motion estimation, magnetic resonance imaging, nonlinear dynamic state estimation, regional wall motion abnormality

## Abstract

Objective: This study investigates the estimation of three dimensional (3D) left ventricular (LV) motion using the fusion of different two dimensional (2D) cine magnetic resonance (CMR) sequences acquired during routine imaging sessions. Although standard clinical cine CMR data is inherently 2D, the actual underlying LV dynamics lies in 3D space and cannot be captured entirely using single 2D CMR image sequences. By utilizing the image information from various short-axis and long-axis image sequences, the proposed method intends to estimate the dynamic state vectors consisting of the position and velocity information of the myocardial borders in 3D space. Method: The proposed method comprises two main components: tracking myocardial points in 2D CMR sequences and fusion of multiple trajectories correspond to the tracked points. The tracking which yields the set of corresponding temporal points representing the myocardial points is performed using a diffeomorphic nonrigid image registration approach. The trajectories obtained from each cine CMR sequence is then fused with the corresponding trajectories from other CMR views using an unscented Kalman smoother (UKS) and a track-to-track fusion algorithm. Results: We evaluated the proposed method by comparing the results against CMR imaging with myocardial tagging. We report a quantitative performance analysis by projecting the state vector estimates we obtained onto 2D tagged CMR images acquired from the same subjects and comparing them against harmonic phase estimates. The proposed algorithm yielded a competitive performance with a mean root mean square error of 1.3±0.5 pixels (1.8±0.6 mm) evaluated over 118 image sequences acquired from 30 subjects. Conclusion: This study demonstrates that fusing the information from short and long-axis views of CMR improves the accuracy of cardiac tissue motion estimation. Clinical Impact: The proposed method demonstrates that the fusion of tissue tracking information from long and short-axis views improves the binary classification of the automated regional function assessment.

## Graphical abstract

We present the estimation of 3D left ventricular (LV) motion using the fusion of different 2D cine magnetic resonance (CMR) sequences acquired during routine imaging sessions. We show that fusing the information from short and long-axis views of CMR improves the accuracy of cardiac tissue motion estimation.

## I. Introduction

The assessment of the regional left ventricular (LV) function plays an important role in the diagnosis of coronary heart disease [1]–[4]. Cardiac magnetic resonance (CMR) imaging offers a non-invasive solution to capture the LV function. Manual assessment of the LV function over numerous images produced by the CMR is tedious, time-consuming and expensive. Many automated methods to quantitatively assess the LV regional function have been proposed in the literature [1]–[4]. However, existing methods utilized only two-dimensional (2D) short-axis sequences of the CMR in generating regional function measurements. Due to high inter-slice distance^1^ between the short-axis sequences acquired along multiple positions of the LV, these 2D sequences do not entirely capture the LV dynamics that lie in 3D space and consist of a complex combination of movements. Due to lack of through-plane information, standard single view 2D CMR sequences are inadequate for estimating the LV dynamics in 3D space. One can overcome this limitation by exploiting the data from other image sequences that are orthogonal to obtain 3D dynamics and an accurate assessment of the LV function.^1^Typically the slice distance is 8 – 10 mm in standard clinical acquisitions.

**FIGURE 1.**
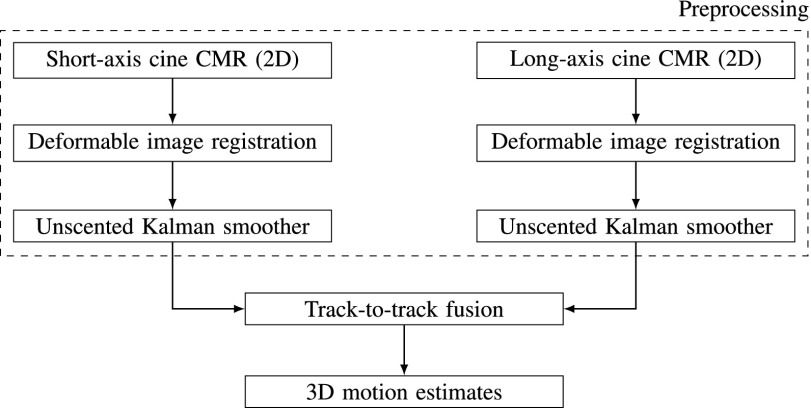
The overall system diagram showing the components of the proposed 3D motion estimation algorithm that fuses information from short-axis and long-axis CMR sequences.

This study proposes a novel approach to fuse motion information available in multiple 2D image views, thereby, utilizing the information available in different CMR short-axis sequences as well as long-axis sequences such as 2-chamber, 3-chamber and 4-chamber views. Fig. 1 depicts the overall system diagram of the proposed method which consists of a preprocessing component and a track-to-track fusion algorithm. The multiview fusion approach proposed in this study differs fundamentally from the earlier methods in the literature which, among others, are based on 3D harmonic phase [5], deformable models [6], [7], incompressible models [8] or image registration of short-axis and long-axis sequences [9]. One of the primary benefits of the proposed method is that it uses only anatomical cine sequences that are commonly used in standard clinical examinations due to their shorter processing times. In contrast, previous methods relied on scanning protocols such as myocardial tagging [5], [6] that require longer scan times, or displacement encoding with stimulated echoes (DENSE) CMR images [7] that are not available in routine clinical scans.

The proposed approach first generates a trajectory for each point on the myocardial boundary using a diffeomorphic moving mesh algorithm [10] within each 2D scan, long-axis and short-axis, given delineation of the myocardium on the first frame. The temporal smoothness of the trajectories is obtained by incorporating the prior information corresponds to the LV dynamics via an extension of the nonlinear dynamic state transition model proposed in [11] to 3D. A nonlinear recursive Bayesian framework known as the unscented Kalman smoother (UKS) is proposed to obtain the mean and covariant matrix correspond to each state vector representing the position and velocity of a myocardial point over a cardiac cycle.

The following summarizes the contributions of the current study.

1) A nonlinear state transition model with an augmented dynamic angular frequency component in the state vector is derived by generalizing the model proposed in [12]. We further extend the model to 3D and use the UKS for state estimation.
2) A track-to-track fusion algorithm that is based on maximum-likelihood approach is proposed for combining multiple trajectories. These trajectories are obtained using the UKS from several short-axis and long-axis 2D sequences. To the best of authors’ knowledge, this is the first application of the track-to-track fusion in medical imaging.
3) We conducted two independent comprehensive evaluations in cardiac image analysis to demonstrate the advantages of the proposed fusion: (a) Comparing the state estimates obtained with the proposed method using anatomical cine CMR to those obtained with the well-known harmonic phase (HARP) analysis technique [13], which is the most common method for tagged CMR image analysis; (b) Applying the proposed approach to detect regional LV motion abnormality following the 17-segment model recommended by the American Heart Association [14], and evaluating the automated classification results with the assessments by expert radiologists.

In the first experiment, the HARP was implemented as described by Osman *et al.* in [13]. The HARP algorithm utilizes the Fourier domain analysis of the tagged MR images as opposed to their spatial domain. Previous works on estimating cardiac 3D motion from tagged images using the HARP concept were reported in [5], [15], [16]. In this paper, we evaluate the results of the proposed algorithm quantitatively by projecting 3D state vectors onto 2D tagged CMR images acquired from the same subjects.

In the second experiment, the proposed algorithm is evaluated in terms of its classification ability to detect the regional left ventricular functional abnormality. The evaluation was performed over 480 segments obtained from twenty normal subjects and ten patients with abnormal heart function. The classifier used in the experiment was built based on Shannon’s differential entropy (SDE) values of two features: normalized radial distance and segment-wise volume/area. Quantitative evaluations were performed for classifier *with and without fusion* in terms of area-under-the-curve (AUC) values of receiver operating characteristic (ROC) curves, and the Bhattacharyya distance metric [17].

## II. Preprocessing

### A. Deformable Image Registration

The objective of the deformable registration is to obtain the point correspondence between two adjacent images $T_{k}$ and $T_{k+1}$ (for $k=1,2\ldots,K-1$) defined over $\Omega \subset {\mathbb R}^{2}$ where $k$ and $K$ denote the frame number and the total number of images in a CMR sequence, respectively. Finding the point correspondence between these pair of images can be expressed as the optimization problem given below.

\begin{equation*} \hat {\phi } = \mathrm {arg} \underset {\phi }{\mathrm {opt}}\; E_{L2}(T_{k},T_{k+1},\phi ({x})) \tag{1}\end{equation*}

where ${x}\in \Omega $ is the pixel location, $\phi: \Omega \rightarrow \Omega $ is a transformation function, and $E_{L2}(\cdot)$ is an $L_{2}$-norm based similarity metric. The optimization problem in (1) generally does not lead to a unique solution without additional constraints which can be added by introducing a deformation field with the continuous monitor function $\mu:\Omega \rightarrow {\mathbb R}$ and curl of end velocity field $\gamma:\Omega \rightarrow {\mathbb R}$ as described below.

#### 1) Moving Mesh Generation

The continuous monitor function $\mu ({x})$ constrained by

\begin{equation*} \int _\Omega {\mu } = |\Omega |\tag{2}\end{equation*}

The objective is to obtain a transformation $\phi $ that meets the following condition.

\begin{equation*} J_\phi ({x}) = \mu ({x}) \tag{3}\end{equation*}

A transformation function $\phi $ that satisfies the condition in (3) can be defined as follows.

Step 1: Generate a vector field $\rho ({x})$ which satisfies the following div-curl system
  \begin{align*}& \mathrm {div}\; \rho ({x}) = \mu ({x}) - 1\tag{4a}\\[-0.5em]{}\smash {\left \{{\vphantom {\begin{matrix}.\\.\\.\\.\\ \end{matrix}}}\right.}& \\[-0.5em]& \mathrm {curl}\; \rho ({x}) = \gamma ({x})\tag{4b}\end{align*}
  with Dirichlet boundary condition $\rho ({x}) = 0 \,\forall \, {x}\in \partial \Omega $.
Step 2: Construct a velocity vector field using $\rho ({x})$:
  \begin{equation*} \nu _{t}({x}) = \frac {\rho ({x})}{t+(1-t)\mu ({x})}, \qquad t \in [{0,1}], \tag{5}\end{equation*}
  where $t$ is an artificial time.
Step 3: By introducing a diffeomorphic function $\psi ({x},t)$, solving the following ordinary differential equation
  \begin{equation*} \frac {d \psi ({x},t)}{dt} = \nu _{t}(\psi ({x},t)), \quad t \in [{0,1}], \psi ({x},t=0)= {x},\tag{6}\end{equation*}
  and setting $\phi ({x})=\psi ({x},t=1)$, we can obtain the transformation function. We refer the reader to [10] and [18] for the graphic processor and the central processing unit based implementations and other algorithm details of the moving mesh based nonrigid registration algorithm.

### B. Image Coordinate Transformation

In order to combine cardiac motion information from different image planes such as short-axis and long-axis sequences, we first need to transform image coordinate based location information into a 3D reference space. We compute the *reference coordinate system* using the following equation:

\begin{equation*} \left [{ \begin{array}{c} {\mathsf x}\\ {\mathsf y}\\ {\mathsf z} \end{array} }\right] = \left [{ \begin{array}{ccc} X_ {\mathsf x}\Delta _{i} & Y_ {\mathsf x}\Delta _{j} & S_ {\mathsf x}\\ X_ {\mathsf y}\Delta _{i} & Y_ {\mathsf y}\Delta _{j} & S_ {\mathsf y}\\ X_ {\mathsf z}\Delta _{i} & Y_ {\mathsf z}\Delta _{j} & S_ {\mathsf z} \end{array} }\right] \left [{ \begin{array}{c} i \\ j \\ 1 \end{array} }\right] \tag{7}\end{equation*}

**where:**
Abbreviation: Expansion
$({\mathsf x},\, {\mathsf y},\, {\mathsf z})$: 3D coordinates of the pixel location $(i,j)$ in the image plane in units of mm.
$S_{\mathsf x {\mathsf y} {\mathsf z}}$: *Image Position (Patient)* attribute elements.
$X_{\mathsf x {\mathsf y} {\mathsf z}}$: The elements of the row direction cosine of the *Image Orientation (Patient)* attribute.
$Y_{\mathsf x {\mathsf y} {\mathsf z}}$: The elements of the column direction cosine of the *Image Orientation (Patient)* attribute.
$(i,j)$: Column and row indices to the image plane with zero-indexing.
$(\Delta _{i}, \Delta _{j})$: *Pixel Spacing* in column and row directions in mm.

The attributes such as Image Position, Image Orientation, and Pixel Spacing are obtained from digital imaging and communications in medicine (DICOM) headers.

### C. Temporal Smoothing of the Dataset

#### 1) State Transition Model for Cyclic Dynamics

Let $({\mathsf x}, {\mathsf y}, {\mathsf z})$ be a myocardial point on the 3D reference coordinate system. The point $({\mathsf x}, {\mathsf y}, {\mathsf z})$ corresponds to $(i,j)$ in the image coordinate system and is computed using (7). Let us define a state vector ${\mathbf{s}} = [{\bar {\mathsf x}}\; {\mathsf x}\; {\dot {\mathsf x}}\;{\bar {\mathsf y}}\; {\mathsf y}\; {\dot {\mathsf y}}\; {\bar {\mathsf z}}\; {\mathsf z}\; {\dot {\mathsf z}}\; \omega]^{T}$ that describes dynamics of the corresponding myocardial point. Elements $\bar {\mathsf x}$ and $\dot {\mathsf x}$ denote, respectively, the mean position and the instantaneous velocity over a single heart beat in the ${\mathsf x}$ co-ordinate direction. Similar elements in the ${\mathsf y}$ and ${\mathsf z}$ co-ordinate directions are defined by $\bar {\mathsf y}$, $\dot {\mathsf y}$, $\bar {\mathsf z}$ and $\dot {\mathsf z}$. The parameter $\omega $ denotes the angular frequency.

We exploit heart’s cyclic dynamic property in deriving the state transition model. The discrete-time state transition equation corresponds to the periodic LV tissue dynamics at image frame $k+1$ is given by:

\begin{equation*} {\mathbf{s}}_{k+1} = f_{k}({\mathbf{s}}_{k}) + v_{k} \tag{8}\end{equation*}

where $f_{k}({\mathbf{s}}_{k}) = \mathrm {blkdiag}(F_{k}, F_{k}, F_{k}, 1)$, $\mathrm {blkdiag}(\cdot)$ denotes the block diagonal matrix, and

\begin{equation*} F_{k} = \left [{ \begin{array}{rrr} 1 & 0 & 0\\ 1-\cos (\omega _{k} T) & \cos (\omega _{k} T) & \frac {1}{\omega _{k}}\sin (\omega _{k} T) \\ \omega _{k} \sin (\omega _{k} T) & -\omega _{k} \sin (\omega _{k} T) & \cos (\omega _{k} T) \end{array} }\right]\tag{9}\end{equation*}

The parameter $T$ is the time interval between two adjacent frames, and the proposed approach relied on the heart rate information from the DICOM header and the number of cardiac frames in computing $T$. The noise sequence $\{v_{k}\}$ is assumed to be normal distributed with zero-mean and covariance $Q_{k}$. Dynamic model (8) is an extended version of the 2D model proposed in [12] to 3D space. Further, the state vector is augmented by introducing a dynamic angular frequency component $\omega $ to accommodate the time-varying cyclic motion property of the underlying tissue motion. The measurement equation is given by:

\begin{equation*} z_{k} = H_{k} {\mathbf{s}}_{k} + \eta _{k} \tag{10}\end{equation*}

where $H_{k} = \mathrm {blkdiag}(h,h,h,1)$, $h = [0\;1\;0]$, $\{\eta _{k}\}$ is normally distributed with zero-mean noise sequence with covariance $R_{k} = \mathrm {diag}([r\;r\;r])$, $\mathrm {diag}(\cdot)$ denoting the diagonal matrix. The measurement equation describes the relationship between the hidden state-space vector ${\mathbf{s}}$ and the observable parameters, which in this study, are the 3D locations of the endocardial or tag intersection points estimated on the CMR images. The sequence of observations $z_{k} = [z_{k, {\mathsf x}}\; z_{k, {\mathsf y}}\; z_{k, {\mathsf z}}]^{T}$ for $k=1:K$ corresponding to the point $(i,j)$ on the image coordinate system is obtained by applying the transformation function $\hat {\phi }$ to $(i,j)$ and mapping them into patient-based coordinate system using (7).

#### 2) Unscented Kalman Smoother (UKS)

**FIGURE 2.**
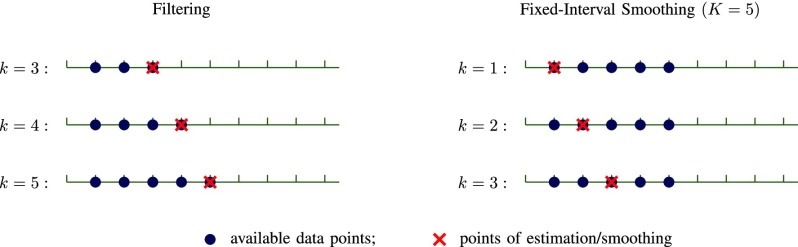
An example to illustrate the conceptual difference between filtering and fixed-interval smoothing. The primary difference is that the filtering estimates rely only on past observations at any given time whereas fixed-interval smoothing estimates are based on observations from both past and future which lead to more accurate results.

The state transition model in (8) is nonlinear and, therefore, we employ the UKS [19] to estimate the state vector $s_{k}$ (for each $k = 1, 2, \ldots, K$). The LV trajectories are estimated as postprocessing which allows for utilizing measurements retrospectively in the estimation algorithm. The UKS used in this study is a retrodiction or smoothing counterpart of the prediction algorithm, unscented Kalman filter (UKF) [20]. In the UKF approach, second-order properties of the underlying probability distributions of the state vector are propagated using a small number of deterministically-selected sample points. This allows for a low computational cost comparable to the extended Kalman filter [21] while providing sufficient estimation accuracy for many nonlinear filtering applications. The primary procedural difference between UKS and UKF is that the UKF uses only the set of observations $z_{1:k}$ to estimate the state ${\mathbf{s}}_{k}$ at frame $k$ while UKS utilizes both $z_{1:k}$ and $z_{k+1:K}$ to get the state estimate, where $K$ is the total number of frames in the CMR sequence. The performance of the filtering algorithm is improved by the smoothing step that utilizes additional measurement in the UKS. There are several variants of smoothing approach, and in this study, we used *fixed-interval smoothing*, the most common type. The conceptual difference between filtering and fixed-interval smoothing is presented in Fig. 2.

## III. Track-to-Track Fusion

The objective of track-to-track fusion is to fuse the state estimates ${\mathbf{s}}^{s}$ and ${\mathbf{s}}^{l}$ corresponding to the same tissue obtained from short- and long-axis image sequences using UKS, respectively. The state estimates obtained using the UKS consist of the mean and the associated covariance values. In this study, we propose to use a maximum likelihood criterion for the track-to-track fusion [22]. Given ${\mathbf{s}}^{s}$ and ${\mathbf{s}}^{l}$, the likelihood function can be defined as follows.

\begin{align*} L({\mathbf{s}})=&-\ln p({\mathbf{s}}^{s}, {\mathbf{s}}^{l}| {\mathbf{s}}) \\&\propto \left ({\left [{\begin{array}{c} {\mathbf{s}}^{s}\\ {\mathbf{s}}^{l}\end{array}}\right] - \left [{\begin{array}{c}I\\ I \end{array}}\right] {\mathbf{s}}}\right)^{T} \Sigma ^{-1} \left ({\left [{\begin{array}{c} {\mathbf{s}}^{s}\\ {\mathbf{s}}^{l}\end{array}}\right] - \left [{\begin{array}{c}I\\ I \end{array}}\right] {\mathbf{s}}}\right)\tag{11}\end{align*}

where the covariance $\Sigma $ is given by

\begin{equation*} \Sigma = \left [{\begin{array}{cc}\Sigma ^{s} & \Sigma ^{sl}\\ \Sigma ^{ls} & \Sigma ^{l}\end{array}}\right]\tag{12}\end{equation*}

The maximum likelihood solution

\begin{equation*} {\mathbf{s}}^{ML} = \arg \max _{\mathbf{s}} L({\mathbf{s}}),\tag{13}\end{equation*}

is then computed by solving $\nabla _ {\mathbf{s}} L({\mathbf{s}}) = 0$. This yields:

\begin{equation*} {\mathbf{s}}^{ML} = \left ({[I\:\, I]\,\Sigma ^{-1} \left [{\begin{array}{c}I \\ I\end{array}}\right] }\right)^{-1}[I\:\, I]\,\Sigma ^{-1} \left [{\begin{array}{c} {\mathbf{s}}^{s}\\ {\mathbf{s}}^{l}\end{array}}\right] \tag{14}\end{equation*}

Define ${\mathcal U}=\Sigma ^{s}$, ${\mathcal V}=\Sigma ^{sl}$ and ${\mathcal W}=\Sigma ^{l}$. Apply inversion of a partitioned matrix:

\begin{equation*} \Sigma ^{-1} = \left [{\begin{array}{cc} {\mathcal U}& {\mathcal V}\\ {\mathcal V}^{T} & {\mathcal W}\end{array}}\right]^{-1} = \left [{\begin{array}{cc} {\mathcal Q}& {\mathcal R}\\ {\mathcal R}^{T} & {\mathcal S}\end{array}}\right] \tag{15}\end{equation*}

where

\begin{align*} {\mathcal Q}=&({\mathcal U}- {\mathcal V} {\mathcal W} ^{-1} {\mathcal V}^{T})^{-1} \tag{16}\\ {\mathcal R}=&- {\mathcal Q} {\mathcal V} {\mathcal W}^{-1} \tag{17}\\ {\mathcal S}=&{\mathcal W}^{-1}+ {\mathcal W}^{-1} {\mathcal V}^{T} {\mathcal Q} {\mathcal V} {\mathcal W}^{-1} \tag{18}\end{align*}

Substitute for $\Sigma ^{-1}$ in (14):

\begin{align*} {\mathbf{s}}^{ML}=&({\mathcal Q}+ {\mathcal R}^{T} + {\mathcal R}+ {\mathcal S})^{-1}({\mathcal Q}+ {\mathcal R}^{T}) {\mathbf{s}}^{s} \\&+ ({\mathcal Q}+ {\mathcal R}^{T} + {\mathcal R}+ {\mathcal S})^{-1}({\mathcal R}+ {\mathcal S}) {\mathbf{s}}^{l}\tag{19}\end{align*}

Substitute ${\mathcal Q}$, ${\mathcal R}$ and ${\mathcal S}$ from (16)–(18) and apply matrix inversion lemma:

\begin{align*} {\mathbf{s}}^{ML}=&(C-B^{T})({\mathcal U}+ {\mathcal P}- {\mathcal V}- {\mathcal V}^{T})^{-1} {\mathbf{s}}^{s} \\&+ ({\mathcal U}- {\mathcal V})({\mathcal U}+ {\mathcal W}- {\mathcal V}- {\mathcal V}^{T})^{-1} {\mathbf{s}}^{l} \tag{20}\end{align*}

Substitute $\Sigma ^{s}$, $\Sigma ^{sl}$ and $\Sigma ^{l}$ in (20):

\begin{align*} {\mathbf{s}}^{ML}=&(\Sigma ^{l}-\Sigma ^{ls})(\Sigma ^{s}+\Sigma ^{l}-\Sigma ^{sl}-\Sigma ^{ls})^{-1} {\mathbf{s}}^{s} \\&+ (\Sigma ^{s}-\Sigma ^{sl})(\Sigma ^{s}+\Sigma ^{l}-\Sigma ^{sl}-\Sigma ^{ls})^{-1} {\mathbf{s}}^{l} \tag{21}\end{align*}

We can simplify (21) using the assumption that cross-covariance values $\Sigma ^{sl}$ and $\Sigma ^{ls}$ between short-axis and long-axis observations are equal to zero:

\begin{equation*} {\mathbf{s}}^{ML} = \Sigma ^{l}(\Sigma ^{s}+\Sigma ^{l})^{-1} {\mathbf{s}}^{s} + \Sigma ^{s}(\Sigma ^{s}+\Sigma ^{l})^{-1} {\mathbf{s}}^{l}\tag{22}\end{equation*}

## IV. Results

We performed two independent comprehensive experimental studies to prove the benefits of the proposed fusion approach. In the first experiment, we report a quantitative performance analysis by projecting the state vector estimates we obtained onto 2D tagged CMR images acquired from the same subjects and comparing them against harmonic phase estimates. In the second experiment, we applied to the proposed method to detect regional LV functional abnormality following the 17-segment model recommended by the American Heart Association [14] and compared the results with ground truth classifications by expert radiologists.

### A. Comparisons with Tagged CMR

**FIGURE 3.**
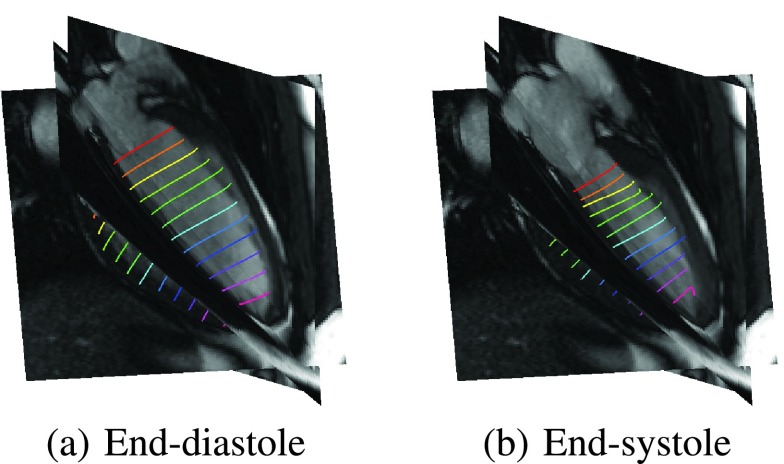
Representative examples showing fusion estimates by the proposed approach plotted against long-axis CMR images.

The data consists of 350 cine and 118 tagged CMR image sequences acquired from 30 subjects (22 males, 8 females; mean age: 55.4±14.4 years; age range: 15 to 78 years). The subjects had various underlying cardiac conditions including dilated cardiomyopathy, aortic valve regurgitation, apical aneurysm, myocardial infarction, atrial fibrillation, cardiac sarcoidosis, myocarditis and pericarditis. The images are two dimensional, and they were acquired on various 1.5T CMR scanners using steady-state free precession, such as TrueFISP, protocols. The following parameters are used for the acquisition: echo time = 1.15 – 1.58 ms; repetition time = 28.0 – 53.2 ms; image size $=(150 \times 192) - (256 \times 248)$; in-plane pixel spacing $= (1.09 \times 1.09) - (1.98 \times 1.98)$ mm; flip angle = 50° – 69°. Fig. 3 shows representative samples of the fusion results at the end-diastolic and end-systolic phases of the cardiac cycle plotted against 2-chamber, 3-chamber and 4-chamber cine CMR sequences.

We used the root mean squared error (RMSE) to compare the estimates by the proposed method using cine CMR sequences and the estimates by the HARP using the tagged CMR sequences. The estimates using the proposed method were three dimensional and, therefore, we had to re-project the estimates onto the corresponding tagged CMR images when calculating the RMSE. We used only the first half of the cardiac cycle since the quality of the tag lines becomes poor in the second half of the cardiac cycle, and produces less reliable HARP estimates in our comparison. Further, we used the middle point of the endocardial and epicardial boundary points for comparison as HARP provides more accurate estimates inside the myocardium rather than the boundary.

**FIGURE 4.**
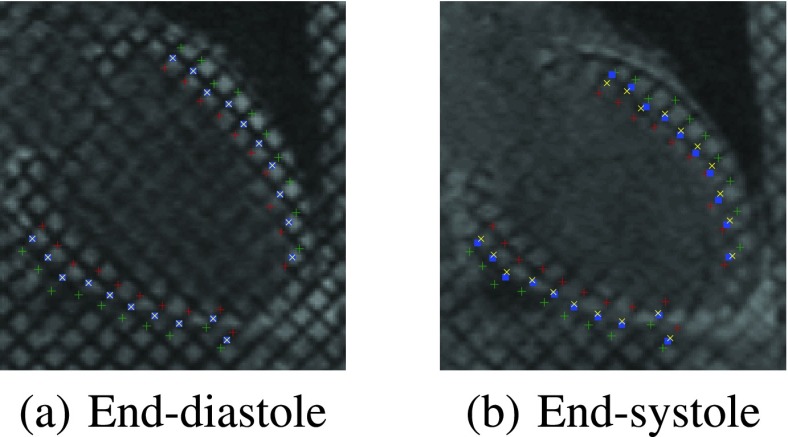
Comparison of the proposed and HARP estimates at end-systolic and end-diastolic phases of the cardiac cycle in 2-chamber view. Green, red and yellow markers, respectively, denote the epicardium, endocardium and corresponding middle points of the projected estimates obtained using the proposed method and cine CMR images; Blue markers indicate the HARP estimates based on tagged CMR images.

**FIGURE 5.**
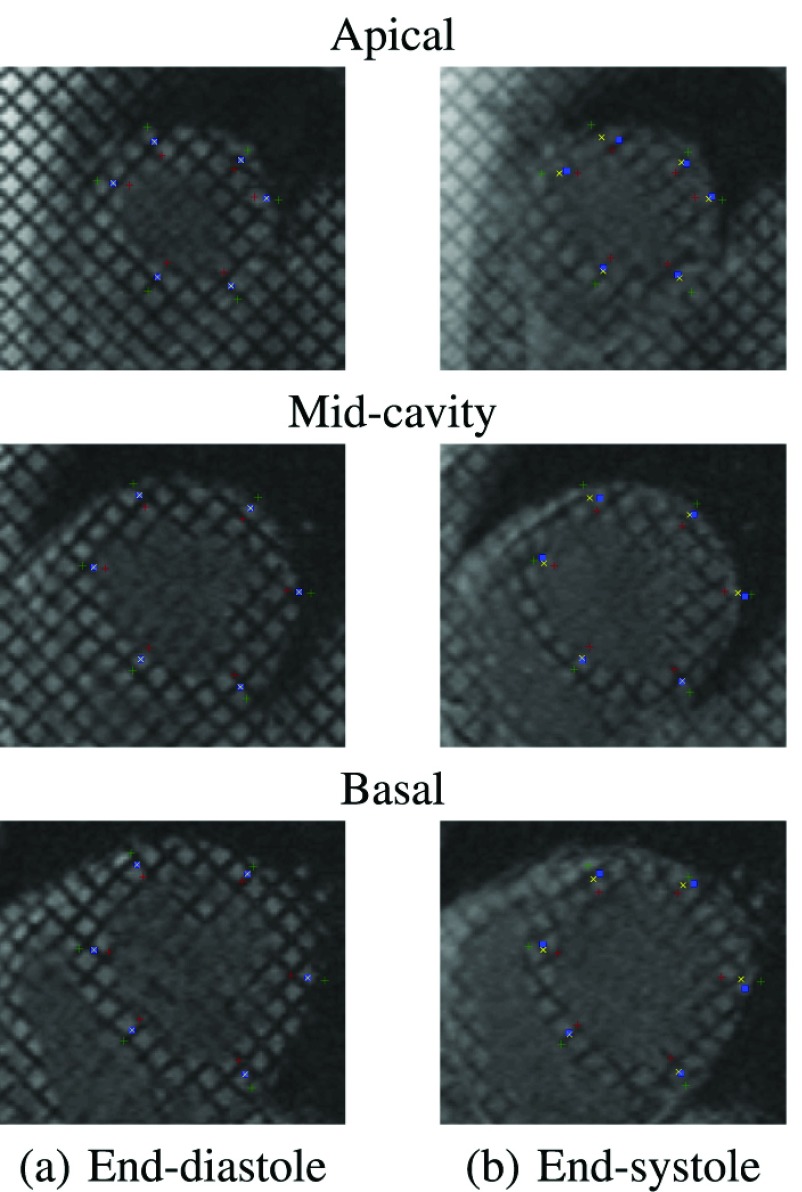
Comparison of the proposed and HARP estimates at end-systolic and end-diastolic phases of the cardiac cycle in apical, mid-cavity and basal short-axis views. Green, red and yellow markers, respectively, denote the epicardium, endocardium and corresponding middle points of the projected estimates obtained using the proposed method and cine CMR images; Blue markers indicate the HARP estimates based on tagged CMR images.

Fig. 4 shows representative sample comparisons between the proposed and HARP estimates on a 2-chamber long-axis sequence. Green, red and yellow markers, respectively, denote the epicardium, endocardium, and corresponding middle points of the projected estimates obtained using the proposed method and cine CMR images; Blue markers denote the HARP estimates based on tagged CMR images. A similar comparison on the short-axis images at apical, mid-cavity and basal slices are given in Fig. 5. The RMSE over $\mathsf N$ number of points is given by, $\mathrm {RMSE} =\sqrt {\frac {1}{\mathsf N} \sum ^{\mathsf N}_{i=1}{({\hat {\xi }}_{i}-{\tilde {\xi }}_{i})^{2}+({\hat {\phi }}_{i}-{\tilde {\phi }}_{i})^{2}}}\vphantom {{\sqrt {\frac {1}{\mathsf N} \sum ^{\mathsf N}_{i=1}{({\hat {\xi }}_{i}-{\tilde {\xi }}_{i})^{2}+({\hat {\phi }}_{i}-{\tilde {\phi }}_{i})^{2}}}}_{j}^{'}}$, where $(\hat {\xi }_{i},\hat {\phi }_{i})$ is the projection of a point on the 2D image coordinate estimated using the proposed method and $(\tilde {\xi }_{i},\tilde {\phi }_{i})$ is the corresponding point by the HARP using tagged CMR images. Table 1 reports the mean and standard deviation of the overall RMSE of the proposed method and the methods without fusion using only short or long axis images, over 114 image sequences. These RMSE values are calculated based on projections onto short as well as long axis tagged CMR images. The $p$-values for unpaired t-tests are also reported in the table. The mean RMSE for the proposed method is equal to 1.3 pixels (1.8 mm).

**TABLE 1:** Comparison of the Overall RMSE (Mean and Standard Deviation) for the Methods With and Without ($i.e.$, Using Only the Short-Axis (SA) or Long-Axis (LA) View) Track-to-Track Fusion. The RMSE was Computed by Projecting the State Estimates Onto SA and LA Tagged CMR Images and Comparing Them Against Harmonic Phase Estimates. The $p$ -Values for Unpaired T-Tests are also Reported. The Proposed Multiview Fusion Method Achieved an RMSE of 1.3 Pixels in Comparison With the HARP Estimates

	Proposed method	SA only	LA only	Proposed vs SA	Proposed vs LA
RMSE (pixels)	1.3 ± 0.5	1.6 ± 0.7	1.7 ± 0.5	$p = 0.0296$	$p< 0.001$
RMSE (mm)	1.8 ± 0.6	2.1 ± 0.9	2.3 ± 0.7	$p = 0.1093$	$p< 0.001$

Table 2 reports the RMSE errors evaluated by projecting onto short-axis and long-axis tagged CMR images separately in first and second rows, respectively, as well as the corresponding $p$-values for unpaired t-tests. Table 2 demonstrate that the proposed method yielded better estimates than the methods without fusion in comparisons to both short and long axes tagged CMR sequences.

**TABLE 2:** Comparisons of the RMSE (Mean and Standard Deviation) for the Methods With and Without ($i.e.$, Using Only the Short-Axis (SA) or Long-Axis (LA) View) Track-to-Track Fusion. The RMSE is Computed Separately by Projecting the State Estimates Onto Short (First Row) and Long (Second Row) Axis Tagged CMR Images and Comparing them Against Harmonic Phase Estimates. The $p$ -Values for Unpaired T-Tests are also Reported. The Proposed Method Yielded Better Estimates Than the Methods Without Fusion in Comparisons to Both Short and Long Axes Tagged CMR Sequences

	Proposed Method	SA only	LA only	Proposed vs SA	Proposed vs LA
RMSE (evaluated on SA tags)	1.6 ± 0.5 mm	1.8 ± 0.7 mm	2.2 ± 0.6 mm	$p=0.3875$	$p< 0.001$
RMSE (evaluated on LA tags)	2.4 ± 0.6 mm	3.2 ± 0.9 mm	2.5 ± 0.6 mm	$p=0.0169$	$p=0.9997$

### B. Regional Abnormality Analysis

**FIGURE 6.**
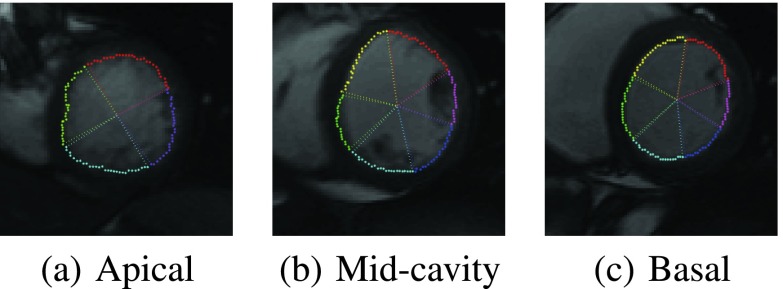
Representative example showing the results of the regional myocardial segmentation using the proposed approach. Following the recommendation by AHA [14], the myocardium was delineated into four, six and six regional segments for apical, mid-cavity and basal slices, respectively.

The regional abnormality analysis was performed over datasets acquired from 30 subjects (20 males, 10 females; mean age: 48.7 ± 15.9 years; age range: 16 – 77 years). The study population composed of 20 normal subjects and 10 patients with abnormal heart function, who had various cardiac diseases including myocardial infarction, ventricular tachycardia, LV pseudoaneurysm, apical aneurysm, cardiac perforation, ascending aortic aneurysm and pericardial effusion. The images were acquired using 1.5T CMR scanners using fast imaging employing steady state acquisition (FIESTA) protocol. The following parameters are used for the acquisitions: echo time = 0.98 – 1.90 ms; repetition time = 2.69 – 4.49 ms; image size $= (256 \times 256)$; in-plane spacing $= (0.66 \times 0.66) - (1.56 \times 1.56)$ mm; flip angle = 45° – 55°. Three representative slices were selected from apical, mid-cavity and basal segments of the LV and the evaluation was performed over $30\times 3$ short-axis image sequences. The regional endocardial borders were automatically segmented following the 17-segmented model recommended by the AHA [14], given the anatomical landmarks, *i.e.*, insertion points denoting the septal wall, on the first frame. A representative example showing the regional segmentation results from apical, mid-cavity and basal short-axis images is given in Fig. 6(a), (b) and (c). In total 480 myocardial segments were analyzed and the classification results correspond to the automated methods were compared with ground truth classifications by radiologists. The manual ground truth classification was built by two experienced radiologists who annotated different portions of the dataset. Binary classification of the motion was used in the evaluation, and a segment was classified as abnormal if the segment is hypokinetic, akinetic, or dyskinetic.

Two independent criteria were used in the evaluation of the discriminative power of each classifier features: 1) the ROC curves [23] with corresponding AUC [24] values; and 2) Bhattacharyya measure [17]. We also measured the performance of the classifier features using accuracy, sensitivity and specificity. We utilized a *leave-one-subject-out* method in the evaluation.

#### 1) Roc and AUC

**FIGURE 7.**
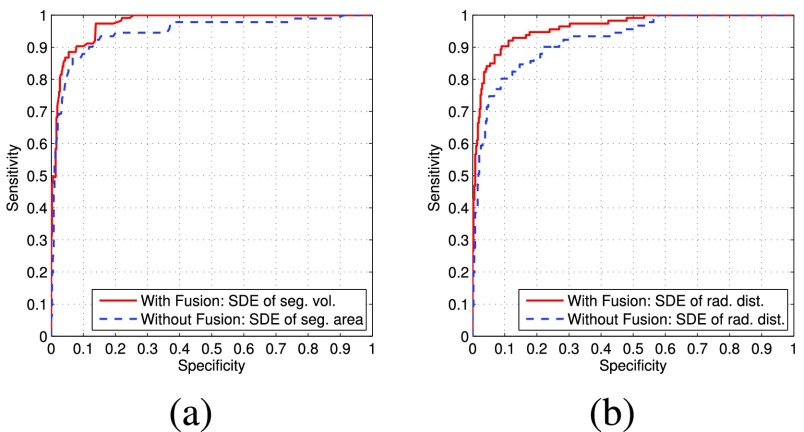
Receiver operating characteristic curves of classifier features for the methods with and without (short-axis sequences only) fusion. The proposed method based on fusion demonstrated better classification ability in both classifier features.

Fig. 7(a) and (b) shows the ROC curves for classifier feature SDE values of segment area/volume and radial distance, respectively. The corresponding AUC values are reported in Table 4. The evaluations based on the ROC curves and the corresponding AUC values for regional cardiac function show that the proposed fusion approach significantly enhances the discriminative ability of each classifier feature over the method that relies only on short-axis sequences in distinguishing the abnormal function from normal function.

**TABLE 3:** Comparison of the Classification Accuracy for the Methods With and Without Track-to-Track Multiview Fusion Using a Leaving-One-Subject-Out Approach. The Method Without Fusion Relied Only on Short-Axis Image Sequences [11]. With Fusion, the Proposed Method Outperformed the Earlier Approach and Yielded an Overall Classification Accuracy of 91.9%

	Proposed method			SA only [11]		
	Acc (%)	Sen (%)	Spe (%)	Acc (%)	Sen (%)	Spe (%)
Apical	90.8	96.9	88.6	92.5	90.0	93.3
Mid-cavity	95.0	95.3	94.9	93.3	93.1	94.1
Base	89.4	97.4	87.3	87.2	100.0	84.9
Overall	91.9	96.5	90.5	90.8	94.5	90.0

Acc=Accuracy, Sen=Sensitivity, Spe=Specificity.

**TABLE 4:** Comparison of the Classification Ability of the Methods With and Without Fusion Assessed in Terms of the Area Under the ROC Curve and Bhattacharyya Distance Metric. The Method Without Fusion Relied Only on Short-Axis Image Sequences [11]. With Fusion, the Proposed Approach Demonstrated Improved Classification Ability in Detecting LV Functional Abnormality

	Proposed method		SA only [11]	
	AUC	${\mathbb B}$	AUC	${\mathbb B}$
SDE of segment area	97.1	0.74	94.3	0.66
SDE of radial distance	95.9	0.70	92.1	0.61

#### 2) Bhattacharyya Measure

We used Bhattacharyya distance metric to evaluate the overlap between the normal and abnormal functions for each classifier feature. The Bhattacharyya distance metric between the distributions $f_{N}$ and $f_{A}$ correspond to the normal function and the abnormal function, respectively, is computed as follows.

\begin{equation*} {\mathbb B} = \sqrt {1-\sum _{r\in {\mathbb R}}\sqrt {f_{N}(r)f_{A}(r)}}, \tag{23}\end{equation*}

A higher ${\mathbb B}$ indicates the better discriminative ability of the classifier as it is associated with lesser overlap between the distributions. Table 4 also reports the Bhattacharyya distance metric values for each classifier feature, which demonstrate the proposed fusion approach leads to improved classification ability for each classifier feature in comparison to the method that relies only on short-axis image sequences.

#### 3) Classification Performance

**FIGURE 8.**
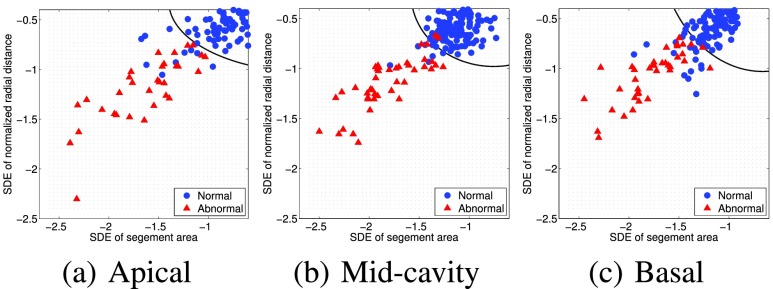
The decision boundaries computed using a Bayesian classifier to detect the normal and abnormal regional myocardial functions based on classifier features. The boundaries were computed separately for apical, mid-cavity and basal slices.

We constructed a nonlinear classification boundary using a naive Bayes classifier approach [25] to utilize the SDE values of both classifier features, the segment area/volume and normalized radial distance in distinguishing normal and abnormal regional heart functions. Fig. 8 depicts the separate decision boundaries corresponds to regional segments from apical, mid-cavity and basal slices. The blue circles and red triangles denote the normal and abnormal regional functions, respectively. The classification performance was measured in term of accuracy, sensitivity and specificity using a *leaving-one-subject-out* strategy. Table 3 reports the classification performance results correspond to the proposed method and the method that uses only short-axis images [11].

The proposed method *with multiview fusion* yielded an overall accuracy of 91.9%, a sensitivity of 96.5%, and a specificity of 90.5%, whereas the method that uses only short-axis image sequences [11] yielded an accuracy of 90.8%, a sensitivity of 94.5%, and a specificity of 90.0%. The evaluation demonstrates the proposed fusion method improves the regional function classification accuracy significantly.

The proposed method took an average of 145 seconds to produce the results for a typical dataset with 10 short-axis and 3 long-axis sequences on an Intel Core i7 CPU with 16GB random access memory. The proposed method requires manual delineation of the LV on the first frame in each sequence, and no other manual intervention is needed.

## V. Discussion

With images obtained at different views and times, one of the leading challenges in CMR image analysis is the fusion of information. Most of the current methods [3], [4], [26], [27] overlook information fusion, an approach that can be much more informative than what is obtained from individual input images. The proposed Bayesian track-to-track fusion provides a solution to fuse information from multiple sources by analyzing the reliability of each source. To the best of authors knowledge, this is the first attempt to fuse cine CMR images acquired from short and long axes sequences to advance the regional assessment of the left ventricular functional assessment. There are few studies on 3D cardiac motion estimation [28], [29], they relied on tagged MR sequences and not on the standard anatomical cine CMR, the most widely used clinical CMR sequence for LV functional assessment.

The RMSE values reported in tables 1 and 2 demonstrate that the proposed fusion approach has a better agreement with tag intersection estimates by the HARP method than the methods that rely only on short or long-axis. The $p$-values reported in Table 2 demonstrate that the proposed method performs significantly better than methods that rely only on short or long-axis when evaluated on a different view. This is due to the fact that the methods rely on a single view are incapable of estimating the through-plane motion, which leads to poor performance, whereas the proposed method combines information from different views and incorporates both in-plane and through-plane motions in the motion estimation.

Automated regional assessment of the LV is a challenging problem. Suinesiaputra *et al.* report an accuracy of 63.7% (base), 67.4% (mid-cavity), and 66.7% (apical) for their method when a binary classification of the visual wall motion scoring was used as a reference [3]. Afshin *et al.* report an accuracy of 84.0% (base), 85.7% (mid-cavity) and 89.8% (apical) for their method for similar classification problem [4]. The proposed method with fusion achieved an accuracy of 89.4% (base), 95.0% (mid-cavity) and 90.8% (apical) when binary visual wall motion scoring is used as a reference.

Tracking the normal functioning LV is more challenging due to large deformations and high variations in velocity values than the ventricle with reduced function. However, abnormal motions such as ventricular dyssynchrony might affect the performance of the proposed tracking algorithm due to variations in motion parameters within the cardiac cycle. The datasets used in our study consisted of only three short-axis tagged MRI sequences acquired from a patient with atrial fibrillation, and the proposed method yielded an RMSE of 1.3 ± 0.4 mm. Further studies are warranted to assess the performance of the proposed algorithm on specific cases with severe motion abnormalities.

The proposed method was evaluated against CMR tagging only in terms of RMSE. The proposed approach fuses the motion information from short and long-axis sequences, and therefore, could be used for tracking both endocardial and epicardial boundaries. Future studies will rely on these boundary tracking to compute the regional strain values to compare them against the CMR tagging-based values.

The proposed method applied for regional function assessment classified the cardiac motion as only normal and abnormal (hypokinetic, akinetic and dyskinetic). Out of 480 regional segments used in this study, the normal, hypokinetic, akinetic and dyskinetic motions were observed in 367, 84, 18, and 11 segments, respectively. Due to the low number of available cases for akinetic and dyskinetic motions, the proposed study did not attempt to classify the motion into specific abnormal motions. Future studies will focus on overcoming the class imbalance problem by adding more cases for under-represented classes and attempt to classify the abnormal motions further.

One of the limitations of the proposed approach is that it assumes the patients have not moved between the short and long axes CMR acquisitions. Visual assessments confirmed that this assumption was held for the majority of the patients. In future, we plan to investigate registration approaches to resolve the problem of aligning short-axis and long-axis CMR acquisitions when patients move during the scans.

## VI. Conclusion

In this study, we proposed a track-to-track fusion approach obtain 3D LV motion estimates from different orthogonal 2D image sequences. The proposed approach uses a deformable image registration method as a preprocessing step to obtain initial trajectories for each myocardial point, given the delineation of the LV on the first frame. Then, a UKS based recursive Bayesian framework was utilized to obtain consistency across the temporal frames in each sequence. The proposed approach was evaluated by projecting the 3D state vector on to 2D tagged CMR images. Our method yielded a competitive performance with a mean RMSE of 1.3 pixels in comparison to well-known HARP estimates. In another independent experimental evaluation, we demonstrated the proposed fusion approach could be used to improve the binary classification of the regional LV function in comparison to earlier methods that do not use fusion and relies only on short-axis CMR sequences.
